# Récidive d'une hernie primaire de Jean-Louis Petit: à propos d'un cas

**DOI:** 10.11604/pamj.2019.33.247.18168

**Published:** 2019-07-23

**Authors:** Nathalie Dinganga Kapessa, Prince Muteba Katambwa, Florent Tshibwid Zeng, Tresor Kibangula Kasanga, Yannick Tietie Ben Nduala, Willy Arung Kalau

**Affiliations:** 1 Département de Chirurgie, Cliniques Universitaires de Lubumbashi, Université de Lubumbashi, Province du Haut-Katanga, République Démocratique du Congo

**Keywords:** Hernie, lombaire, récidive, Hernia, lumbar, recurrent

## Abstract

La hernie de Jean-Louis Petit constitue avec la hernie du quadrilatère de Grynfelt le groupe des hernies lombaires. Son diagnostic clinique est confirmé par la tomodensitométrie, et éventuellement l'échographie ou la radiographie. L'indication opératoire est formelle du fait de la tuméfaction ou de la gêne fonctionnelle, mais plus encore de l'étranglement toujours à craindre. Nous rapportons un cas rare de récidive de hernie primaire de Jean Louis Petit, chez un homme de 65 ans.

## Abstract (en)

Hernia of the Jean Louis Petit triangle and hernia of Grynfeltt’s quadrilateral space can be classified as lumbar hernias. Its clinical diagnosis is confirmed by computed tomography and, possibly, by ultrasound or x-ray. There is a formal indication for surgery including swelling or functional discomfort but, above all, the risk of strangulation. We here report a rare case of recurrence of primary hernia of the Jean Louis Petit triangle in a 65-year old man.

## Introduction

La hernie de Jean-Louis Petit est une hernie lombaire caractérisée par la protrusion de l'omentum ou l'intestin dans le triangle lombaire inférieur (ou de Jean-Louis Petit). Celui-ci est l'interstice compris entre le bord postérieur de l'oblique externe en avant, le bord antérieur du grand dorsal en arrière et la crête iliaque en bas, qui est la base du triangle. Superficiellement, il est limité par le fascia superficiel et en profondeur par l'oblique interne, avec des fibres du transverse abdominal et de la lame postérieure du fascia lombodorsal [1-4]. Les hernies lombaires constituent moins de 2% de toutes les hernies et seulement 5% d'entre elles sont inférieures, faisant de la hernie de Jean-Louis Petit, une des formes inhabituelles des hernies [1, 3]. Les formes primaires sont rares, mais elles peuvent être mimées par les hernies incisionnelles naissant du flanc à partir d'incisions pour des opérations rénales ou par des incisions faites pour la récolte des greffons osseux sur la crête iliaque [4]. Les petites hernies sont asymptomatiques alors que les plus grosses se présentent comme des masses sensibles donnant des lombalgies. Le diagnostic est confirmé par le CT scan [2, 4]. Naturellement, ces hernies prennent de plus en plus de volume, c'est pour cette raison qu'il est recommandé d'intervenir. La laparoscopie ou la chirurgie ouverte peuvent être faites, en présence d'un large défet. L'utilisation des prothèses donne des meilleurs résultats [2]. Les récidives sont rares [4]. Cet article présente le cas d'un patient traité pour récidive d'une hernie de Jean-Louis Petit.

## Patient et observation

**Figure 1:**
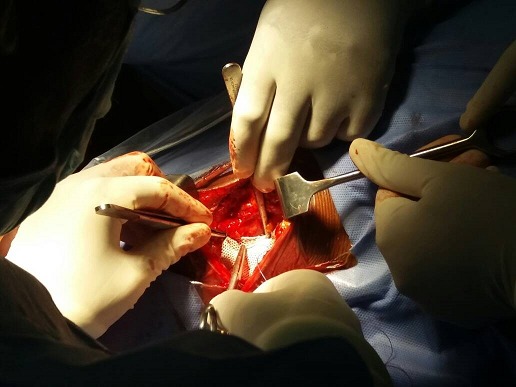
Pose d'une prothèse non résorbable sur la zone de déhiscence du fascia transversalis

Il s'agissait d'un patient âgé de 65 ans qui avait consulté pour une gêne et tuméfaction au flanc droit. Il était cultivateur depuis plusieurs années; opéré pour la même plainte il y avait 3 ans et pour hernie inguinale bilatérale il y avait 31 ans. Il n'y avait pas de notion des vomissements, pas d'arrêt des matières ni des gaz. A l'examen physique, l'etat général était conservé. Une tuméfaction d'environ 15 centimètres de diamètre était située dans la région lombaire droite en regard du triangle de Jean-Louis Petit. La peau en regard comportait une cicatrice d'environ 4 centimètres, oblique en bas et en avant. La masse était molle, à surface lisse, non sensible, réductible, expansive à l'effort (toux, hyperpression abdominale). L'indice de masse corporelle (IMC) calculée était de 16,5 kilogramme/m^2^. L'échographie réalisée avait conclu à une masse lipomateuse en regard du triangle de Jean-Louis Petit. Par manque de moyen, un CT scan n'avait pas été réalisé. Sur base du diagnostic clinique, une herniorraphie avait été indiquée, abordant la masse par une incision oblique en bas et en avant, à environs 4 cm au-dessus de la crête iliaque; le patient étant en décubitus latéral gauche. Le constat après dissection des tissus sous cutanés révélait une graisse retro péritonéale faisant protrusion au travers le triangle de Jean-Louis Petit (hernie extra péritonéale de Thorek). Après excision de la masse graisseuse, la réfection pariétale avait été réalisée par l'insertion d'une prothèse non résorbable placée contre le fascia transversalis et suturée aux muscles limitrophes (Figure 1) Les suites post opératoires immédiates étaient simples, caractérisées par des douleurs de la plaie incisionnelle pour lesquelles le patient avait reçu du paracétamol comprimé à raison de 3 grammes par jour, répartis en 3 prises. Un mois après l'intervention chirurgicale, le patient n'a pas présenté de plainte et à ce jour (une année après), il n'y a pas de récidive Figure 1.

## Discussion

Les hernies lombaires sont des pathologies rares. Depuis la première publication par Garangeot RJC en 1731, seulement environ 300 cas ont été rapportés et il est reconnu depuis 1920 que les hernies lombaires inférieures (ou de Jean-Louis Petit, éponyme en l'honneur du Français qui a décrit l'anatomie chirurgicale lombaire inférieure en 1783) sont les plus fréquentes [5]. Les formes congénitales sont rares (20%) alors que les hernies lombaires inférieures acquises sont largement secondaires [4]. Plusieurs facteurs de risque ont été décrits pour les hernies spontanées, il s'agit entre autres de l'âge, l'obésité, la maigreur extrême, l'amaigrissement intense, une maladie chronique débilitante, l'atrophie musculaire, la bronchite chronique, la plaie infectée et le sepsis post-opératoire [6]. L'âge des patients se situant généralement entre 50 et 70 ans [7], la profession et la maigreur ont été retrouvés dans notre cas. Le mécanisme évoqué serait que la diminution de la graisse favorise la rupture des orifices vasculonerveux qui traversent le fascia dorsolombaire. Les situations augmentant la pression intra-abdominale agiraient comme des facteurs qui déclenchent l'apparition de ces hernies [6]. Plusieurs classifications ont été proposées, y compris celle d'Alfredo Moreno-Egea, qui prend en compte 6 éléments (localisation, taille, contenu, atrophie musculaire, étiologie et récurrence) et proposant les options thérapeutiques pour chacun des 4 types retenus [8]. Le diagnostic clinique de hernie lombaire requiert une grande suspicion clinique. Celle-ci dépend de la taille et du contenu, lequel peut être la graisse rétropéritonéale, le rein ou le colon et plus rarement, l'intestin grêle, l'omentum, la rate, l'ovaire ou l'appendice [7]. Quelques fois, la présentation est semblable à celle d'un lipome. Ce diagnostic doit être exclu devant une masse qui est expansive à la toux et à l'effort intense, habituellement réductible et tendant à disparaitre quand le patient est en décubitus dorsal. En général, le patient se plaint de la tuméfaction et parfois, de douleur au niveau de la tuméfaction. Habituellement, la tuméfaction grossit jusqu'à altérer la symétrie du tronc [6]. Il y a 3 ans, quand le patient avait consulté un centre médical de la place pour une tuméfaction dans la région lombaire, un diagnostic de lipome avait été retenu et pour lequel il aurait subi une exérèse. Ceci rejoint la littérature qui soutient la similarité clinique entre un lipome et la hernie lombaire et delà, attire l'attention du clinicien quant au diagnostic différentiel des tuméfactions lombaires. Ce diagnostic, en dehors d'une hernie peut inclure un lipome, un abcès, un hématome ou une tumeur des parties molles [9]. Lorsque le contenu est intestinal, le bruit qui lui est caractéristique est audible à l'auscultation au stéthoscope. S'il existe en plus une strangulation, s'ajoutent à la symptomatologie, les nausées, le vomissement, l'arrêt des matières et des gaz et la distension abdominale alors qu'à l'examen physique, la tuméfaction devient irréductible [6, 7, 10]. Dans le cas où le contenu est rénal, le patient se présente avec des symptômes urinaires tels que l'hématurie, l'oligurie et des coliques néphrétiques [6].

**Figure 2:**
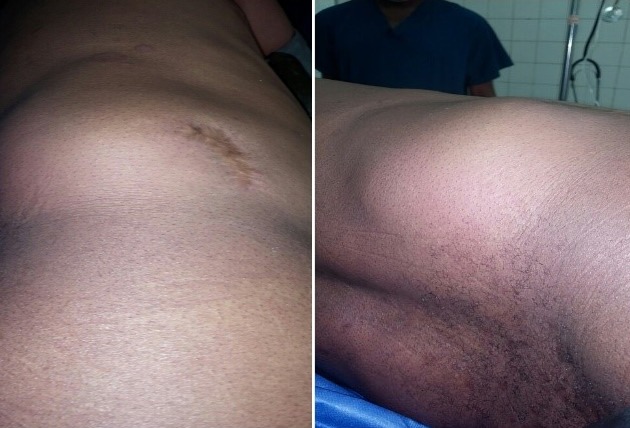
Masse lombaire en regard du triangle de Jean Louis Petit, sur laquelle on voit la cicatrice de la première intervention

Plusieurs investigations paracliniques peuvent être faites selon la présentation clinique. C'est ainsi qu'on peut réaliser des radiographie latérale ou oblique de la région lombaire, qui montreront des anses remplies de gaz et se trouvant en dehors de la cavité abdominale, si le contenu est intestinal. Les radiographies avec produit de contraste de la partie haute du tube digestif aident à délimiter le segment intestinal hernié. L'urographie intraveineuse permet de voir le déplacement rénal ou urétéral dans la hernie. L'échographie ne parvient pas à bien monter la hernie à cause d'un faible indice de suspicion et aussi à cause de la présence de la graisse [7]; ce qui fut le cas pour notre patient. La meilleure évaluation paraclinique reste le CT scan, qui fournit des informations détaillées sur l'anatomie de la région lombaire, l'extension du défect, la présence des viscères dans la hernie. Le CT scan permet de différencier la hernie de l'atrophie musculaire au cours de laquelle il n'existe pas de défect dans le fascia et dont le traitement ne recourt pas à la chirurgie. Il différencie aussi la hernie de l'hématome, de l'abcès et de la tumeur des tissus mous [11]. Par manque de moyen, le patient n'a pu réaliser que l'échographie lombaire, ceci démontre le challenge que représente la prise en charge classique de ce type de maladie en milieu défavorisé. En combinant les éléments cliniques au résultat échographique ayant identifié une masse lipomateuse en regard du triangle de Jean-Louis Petit, nous avons retenu le diagnostic de récidive d'une hernie lombaire inférieure primaire Figure 2. La chirurgie vise la correction du défect et la reconstruction d'une paroi abdominale suffisamment élastique mais résistant aux contraintes physiques journalières. Elle devrait toujours être indiquée compte tenu du risque de strangulation en cas de contenu intestinal [12]. Parmi les différentes techniques décrites, on trouve la fermeture anatomique, le recouvrement de l'aponévrose par des flaps musculofasciaux et la pose des prothèses en maille par voie rétropéritonéale ou laparoscopique transabdominale [6]. L'abord laparoscopique présente certains avantages par rapport à la voie classique, il s'agit notamment d'une plus courte durée d'hospitalisation et de moins de complications post-opératoires. Il existe cependant un plus grand risque de complications per-opératoires [3]. Pour une utilisation plus efficiente des différentes techniques, on peut se référer à la classification proposée par Alfredo Moreno-Egea. Notre patient étant classé dans le Type A, nous avons recouru à une herniorraphie par voie ouverte, par manque de matériels suffisants pour une laparoscopie extra-péritonéale. Nous avons utilisé une prothèse en maille. Celle-ci est actuellement recommandée comme traitement optimal des hernies lombaires unilatérales en plus du fait qu'elle prévient les récidives [12].

## Conclusion

Les chirurgiens ne rencontrent qu'une seule fois un cas de hernie lombaire primaire au cours de toutes leurs carrières [3]. Ceci illustre leur rareté et encore plus, celle des récidives. Dans nos milieux à faible ressource, le chirurgien doit affiner la détection clinique de cette affection, la plupart des patients ne disposant pas de moyens suffisants pour réaliser un CT scan, qui reste le gold standard du diagnostic. Malgré les différentes techniques recommandées, la voie ouverte reste la plus abordable dans notre milieu.

## Conflits d’intérêts

Les auteurs ne déclarent aucun conflit d'intérêts.
